# Cleft Lip Appearance Secondary to Ulcerating Hemangioma

**DOI:** 10.7759/cureus.85584

**Published:** 2025-06-09

**Authors:** Rajarajan Paulpandian, Kota Nithishwar

**Affiliations:** 1 Pediatrics/Neonatology, Sri Venkateshwaraa Medical College Hospital and Research Centre, Pondicherry, IND

**Keywords:** cleft lip, deformity, infantile hemangioma, ulceration, upper lip

## Abstract

Infantile hemangiomas are frequently observed as soft tissue growths during infancy. Although many cases tend to resolve without treatment, some may lead to complications that require prompt management. Here, we present a case of a lip hemangioma in an infant that resembled a cleft lip in the newborn period and resulted in significant early and delayed complications. A 40-day-old female infant was brought with complaints of a reddish raised lesion over her upper lip. The lesion started as a small cleft on day 14 of life, which subsequently increased in size rapidly and ulcerated. She was having feeding difficulty due to pain and not gaining weight. She was admitted with a working diagnosis of complicated infantile hemangioma with ulcer. She was evaluated for internal hemangiomas and syndromic association in view of a large segmental involvement. She was started on oral propranolol along with local wound care and discharged with advice to follow up. The lesion gradually involuted in five months’ time. Currently, she is three years old with sequelae in the form of atrophy, scar, fibrofatty remnant, and loss of lip contour, awaiting plastic surgery. The current case report highlights the rare presentation of an infantile hemangioma ulcerating in the early months of life, resulting in the appearance of a cleft and the need for timely medical intervention before ulceration develops to avoid long-term sequelae and surgery.

## Introduction

The International Society for the Study of Vascular Anomalies (ISSVA) classifies vascular birthmarks into two main categories: vascular tumors and vascular malformations [1]. Vascular tumors are neoplasms arising from the vasculature, which includes infantile hemangioma (IH), tufted angioma, kaposiform hemangioendothelioma, pyogenic granuloma, and hemangiopericytoma. On the other hand, vascular malformations are anomalous blood vessels without any endothelial proliferation, which include capillary, venous, lymphatic, arterial, or combined malformations [2]. Vascular tumors present a few weeks after birth, attain rapid growth in the first year of life, and then slowly involute in childhood, whereas malformations are present at birth, grow commensurately with the child, cannot involute, and have the potential to expand hemodynamically [2]. IH, a vascular tumor, is the most common benign soft tissue tumor of infancy, occurring in around 5% [3]. It is more common in females than males (3:1), Caucasians, those with a low birth weight, and those with decreasing gestational age [4]. The other risk factors of IH are advanced maternal age, multiple gestation, pre-eclampsia, and placenta previa [4]. The most common location is the head and neck region, with the majority of these lesions developing over the central face at the sites of development fusion [5]. There are two classifications of IH: one based on the extent of soft tissue involved (superficial, deep, and mixed) and another based on anatomic configuration (focal and segmental). The focal type arises as a discrete, round lesion from a single point, whereas the segmental type involves a broader area determined by embryonic placodes [6]. The clinical appearance varies based on the depth of the lesions. The superficial type presents as a bright red non-compressible nodule, the deep type presents as a subcutaneous, partially compressible nodule with an overlying bluish hue, and the mixed type has a combination of both [7].

IH goes through three phases of natural evolution: proliferative, plateau, and involutional phases. It appears in the first two to three weeks of life and then proliferates rapidly in the first year of life followed by a slow involution [8]. IH involutes spontaneously in the simple, non-complicated types, but the high-risk types require timely intervention. The risk factors include segmental type including those associated with PHACES (Posterior fossa malformations, Hemangioma, Arterial anomalies, Cardiac anomalies, Eye anomalies and Sternal clefting and Supraumbilical abdominal raphe) and LUMBAR syndromes (Lower body infantile hemangioma, Urogenital anomalies, Ulceration, Malformations of the spinal cord, Bony deformities, Anorectal malformations, Arterial anomalies and Renal anomalies), large size (>5 cm), multiple hemangiomas, and location over vital facial structures such as eye, nose, lip, and lower jaw, which can lead to various complications [9].

IH of the upper lip is usually a mixed, focal-type hemangioma, which has a tendency to rapidly proliferate and slowly involute [10]. These features invariably result in short-term complications such as ulceration, pain, and feeding difficulties, and long-term issues such as disfigurement secondary to scar, atrophy, and fibrofatty remnant [11]. The upper lip generally tends to perform poorly compared to the lower lip, especially if it is large, segmental and the vermilion border is involved, as this tissue is unique and cannot be reconstructed easily [12]. Hence, it is imperative to identify this lesion in the early proliferative phase. We present the case of a child who was identified by her parents to have a visible cleft in the lip by day 14 of life but presented us too late with ulcer and segmental involvement of more than 5 cm including vermilion border on day 40. She required five months of oral propranolol with slower involution causing permanent scar, atrophy, and loss of lip contour.

## Case presentation

**Figure 1:**
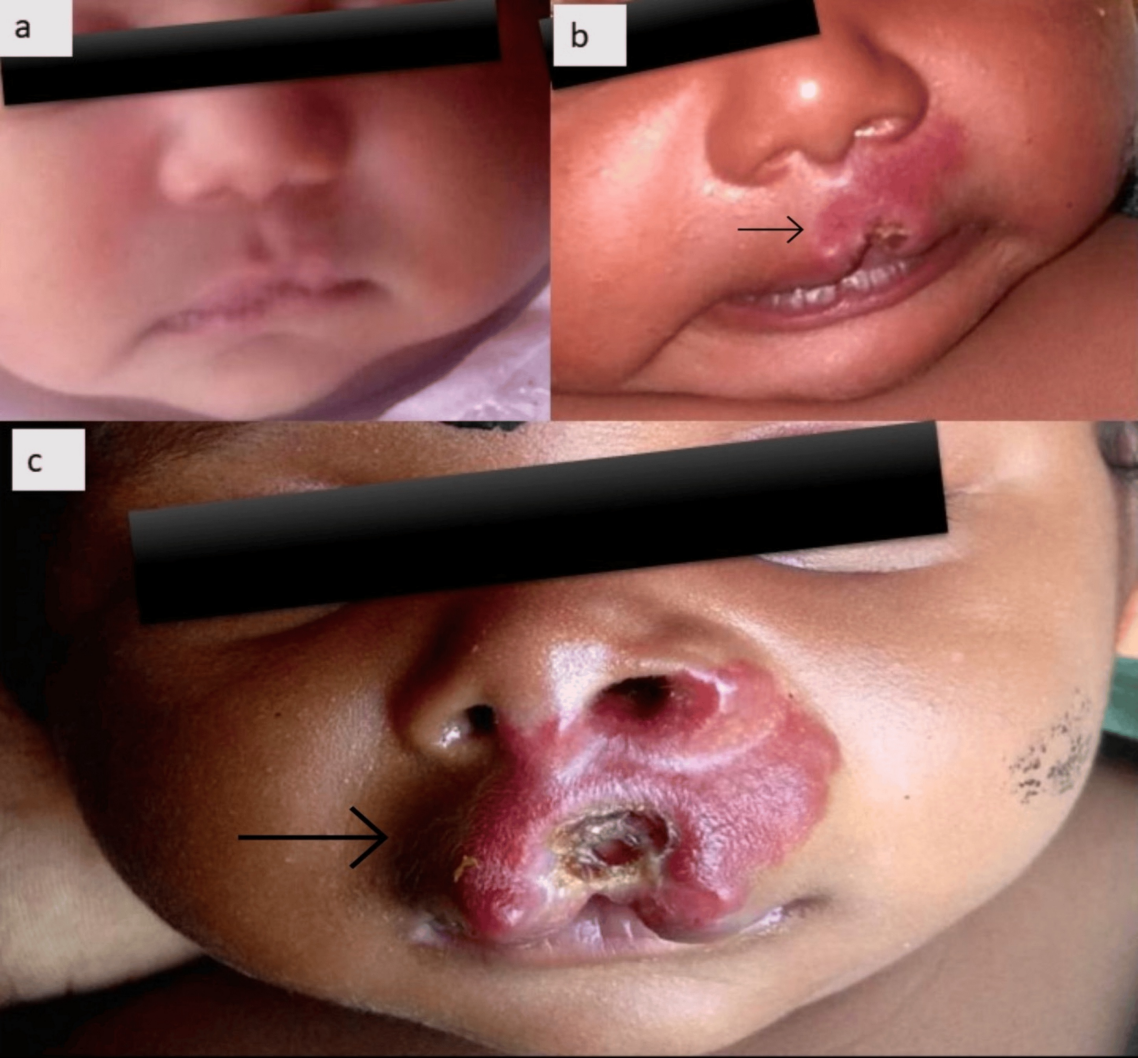
(a) Neonate at birth with normal lip. (b) Infantile hemangioma presenting as a cleft in the lip with surrounding redness in the second week of life. (c) The lesion progressed to a large segmental hemangioma, with ulcer resulting in a cleft lip appearance on presentation to us at day 40 of life.

A 40-day-old female infant was brought by her parents with a complaint of reddish raised lesion over her upper lip. There was no lesion present at birth, and the first lesion was first noticed as a small cleft on day 14 of life, which had increased in size significantly in the next four weeks and presented to us on day 40 of life with an enlarging depression in the center (Figure 1).

She has been having difficulty in feeding since then and not gaining weight adequately. She was born full-term, weighing 3 kg, by spontaneous vaginal delivery to a primigravida mother from a third-degree consanguineous marriage. The antenatal period was uneventful except for preeclampsia, diagnosed a week before delivery requiring oral labetalol. The natal and immediate postnatal periods were uneventful, and she received her birth vaccines. There was no family history of similar lesions. At the time of admission on day 40 of life, the neonate was euthermic with a weight of 3.50 kg (10th centile), length of 50 cm (15-50th centile), and head circumference of 36.5 cm (15-50th centile). Her vitals were normal and were recorded as follows: heart rate of 140 beats/min, respiratory rate of 40 breaths/min with SpO_2_ of 95% in room air, capillary refill of 2 seconds, and normal central and peripheral pulses with blood pressure of 74/50 (58 mm of Hg). Her general physical examination was unremarkable. Systemic examination revealed anterior fontanelle at level with normal tone, normal breath sounds, and first and second heart sounds with no murmur; the abdomen was soft with no organomegaly. On local examination, there was a single well-defined, dull red plaque of size 6x5 cm over the upper lip extending into the left nostril with a central ulceration. With the above findings, the following diagnoses were considered. The provisional diagnosis was IH (vascular tumor) with ulcer manifesting as cleft lip, and a differential diagnosis of vascular malformation was made. The possibility of vascular malformation was kept less likely as the lesion was absent at birth and the presentation of the lesion a few weeks after birth with a rapid growth favored a vascular tumor.

**Figure 2:**
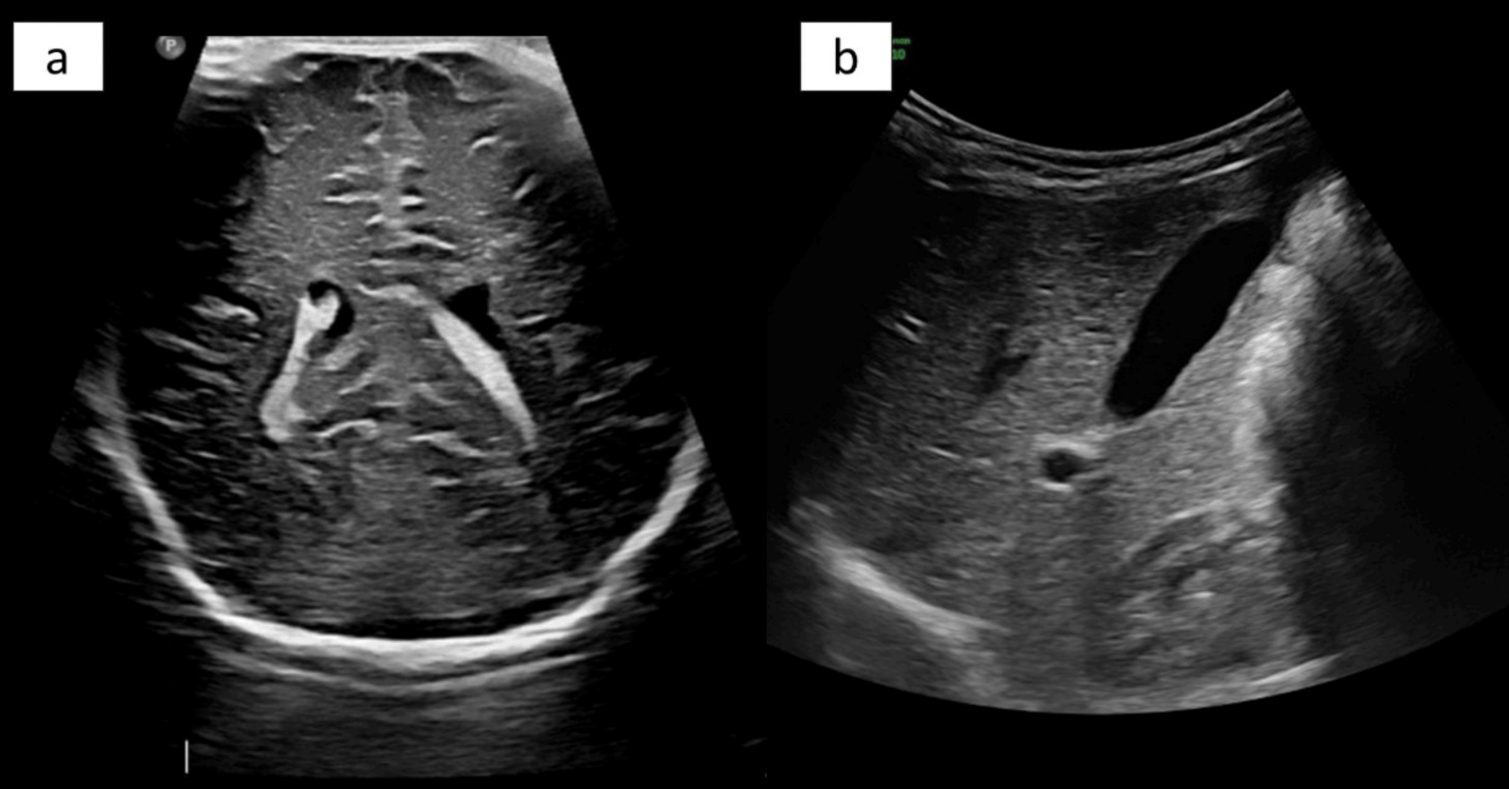
(a) Normal ultrasound of the cranium and (b) ultrasound of the liver of the index case.

The infant was evaluated for internal hemangiomas and PHACES syndrome in view of large segmental facial IH measuring more than 5 cm [13]. Her baseline investigations were within normal limits (Table 1). Ultrasound scans of the cranium and abdomen/liver with Doppler were performed to rule out internal hemangiomas, which were normal (Figure 2). Ophthalmic evaluation and screening echocardiogram were normal. MRI was suggested to rule out intra-cranial anomalies as a part of work-up for PHACES syndrome [14]. However, due to logistic reasons, it could not be done and was deferred.

**Table 1:** Laboratory findings of the index case

Test	Result	Reference range
White blood cells	9 x 10^9^/L	6-14 x 10^9^/L
Neutrophils	56%	54-62%
Lymphocytes	30%	25-33%
Hemoglobin	13 g/dL	10.5-14 g/dL
Platelets	350 x 10^9^/L	84-478 x 10^9^/L
Urea	14 mg/dL	5-18 mg/dL
Creatinine	0.4 mg/dL	0.03-0.50 mg/dL
Sodium	137 mmol/L	134-144 mmol/L
Potassium	4 mmol/L	3.5- 5.6 mmol/L
Chloride	100 mmol/L	98-106 mmol/L
Plasma glucose	4.4 mmol/L	3.3-5.5 mmol/L

**Figure 3:**
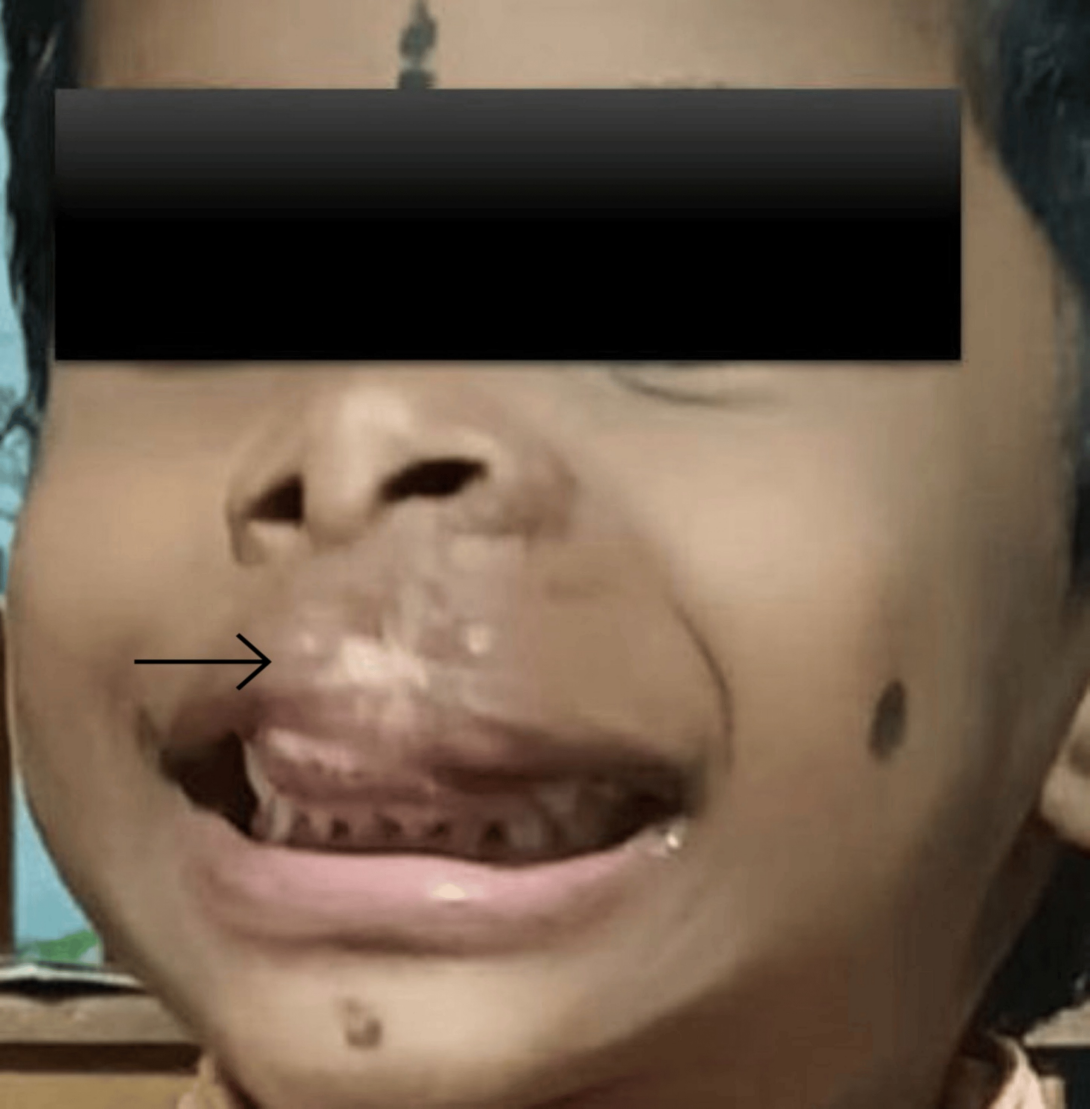
The hemangioma has healed with significant scar, atrophy, and fibrofatty remnant altering the lip contour.

As propranolol needed to be started inpatient in less than eight weeks of age, the infant was admitted and evaluated [15]. Prior to starting propranolol, baseline heart rate, blood pressure, blood sugar, and electrocardiogram were documented to be in the normal range. Oral propranolol was started at a dose of 1 mg/kg/day in two divided doses with monitoring of heart rate and blood pressure. Local wound care in the form of chlorhexidine gauze dressings and topical mupirocin ointment were also started along with paracetamol for pain. As the infant tolerated propranolol well, she was discharged after 48 hours, and parents were advised to have serial visits for monitoring the progress of the lesion and dose titration. Specific advice for the use of propranolol was given, such as timing the drug intake along with feeding, avoiding the drug if the infant is ill, and giving the second dose early in the night with continuation of night feeds [15]. Unfortunately, the parents defaulted the treatment after a week’s time. Subsequently, they were counseled appropriately, following which the compliance improved. They had serial visits, during which the size of hemangiomas was documented and photographed to guide in management, and dose was increased accordingly with monitoring of heart rate, blood pressure, and blood sugar. She required a maximum dose of 3 mg/kg/day. She started feeding well with good weight gain within two weeks of treatment, coinciding with the healing of ulcer. The lesion eventually reached the involution phase (no visible redness with scar formation) after five months of propranolol. Currently, she is three years old and has significant sequelae in the form of scar, atrophy, fibrofatty remnant, and loss of lip contour, awaiting plastic surgery (Figure 3).

## Discussion

The index case had an aggressive hemangioma of the upper lip, with a segmental involvement extending up to the nose. The course was complicated by significant short-term complications such as ulcer, pain, and feeding difficulty, and resulted in permanent disfigurement. Most of the IHs are benign and self-limiting, requiring no active intervention, but a small subset of them can have significant outcomes [16]. It is essential to identify clinical factors associated with functional impairment or life-threatening complications in IHs, as early intervention leads to better outcomes. Segmental IHs are particularly important to recognize, as they are associated with the highest risk of complications and may be linked to syndromic conditions such as PHACES (Posterior fossa anomalies, Hemangioma, Arterial anomalies, Cardiac defects, Eye abnormalities, and Sternal clefting or supraumbilical raphe) and LUMBAR (Lower body hemangioma, Urogenital anomalies, Myelopathy, Bony deformities, Anorectal malformations, and Renal anomalies). The anatomical location of the hemangioma is another critical factor influencing the risk of complications. Perineal hemangiomas are prone to ulceration. Lesions on the fronto-temporal region of the face may be associated with underlying brain anomalies, while mandibular involvement may suggest the presence of congenital cardiac defects. Periocular hemangiomas can result in visual impairment, and nasal tip involvement may lead to nasal deformities. Hemangiomas in the beard distribution area-which includes the preauricular skin, chin, neck, and bilateral lower lip-can indicate a risk of airway obstruction. Involvement of the liver may lead to hepatomegaly, high-output cardiac failure, and consumptive hypothyroidism. Similarly, hemangiomas in the lumbosacral region may be associated with spinal dysraphism, including occult spina bifida [9]. Lesion characteristics also contribute to risk stratification. The presence of five or more cutaneous hemangiomas raises concern for hepatic hemangiomatosis. A lesion size greater than 5 cm typically qualifies as a segmental IH, and each 10 cm² increase in lesion size is associated with a 5-9% increase in the risk of complications. Lesions larger than 25 cm² carry a particularly high risk of ulceration [17]. Patient-related factors include prematurity, low birth weight, and syndromic association [4].

Hemangioma of the lip is of particular concern as it occurs in a prominent facial location. It has a rich vascular supply with large blood vessels approximating at the surface, making it one of the most common locations of IH to ulcerate and bleed [18]. Ulcer, the most common complication of hemangioma, occurs due to increased tissue hypoxia, leading to dermal fibrosis and surface breakdown [10]. The risk of ulceration is higher in lips (18%) compared to the overall risk of ulcer in IHs (15%) [18]. Once the ulcer has formed, it invariably leads to permanent disfigurement with scar formation, atrophy, and loss of lip contour. The location is also significant from the surgical point of view as the vermilion border is unique, found nowhere else in the body, making it imperative to preserve it for a satisfactory cosmetic appearance [19].

The ideal management of a lip hemangioma should be a step-wise process with the initiation of medical treatment early in the proliferative phase before significant ulcer forms. The next step is to wait till complete involution so that partial restoration of anatomy will occur. This allows the full extent of damage to be assessed before undertaking surgical debulking [19]. Wound care and propranolol form the main treatment of ulcerated IH. Pulsed dye laser 595 nm can be used as an adjunctive treatment for ulcerated hemangiomas that fail to improve with wound care and medications [17]. The role of systemic corticosteroids is limited to children with contraindications of propranolol such as cardiogenic shock, significant sinus bradycardia, second- and third-degree heart block, heart failure, bronchospasm, and preterm infants with corrected age <5 weeks [17]. As subjective assessment is the mainstay of management in IH, the use of validated objective tools such as the Hemangioma Activity Score (HAS), which is an easy and quick scoring system, can help clinicians standardize the evaluation and treatment response [20].

**Figure 4:**
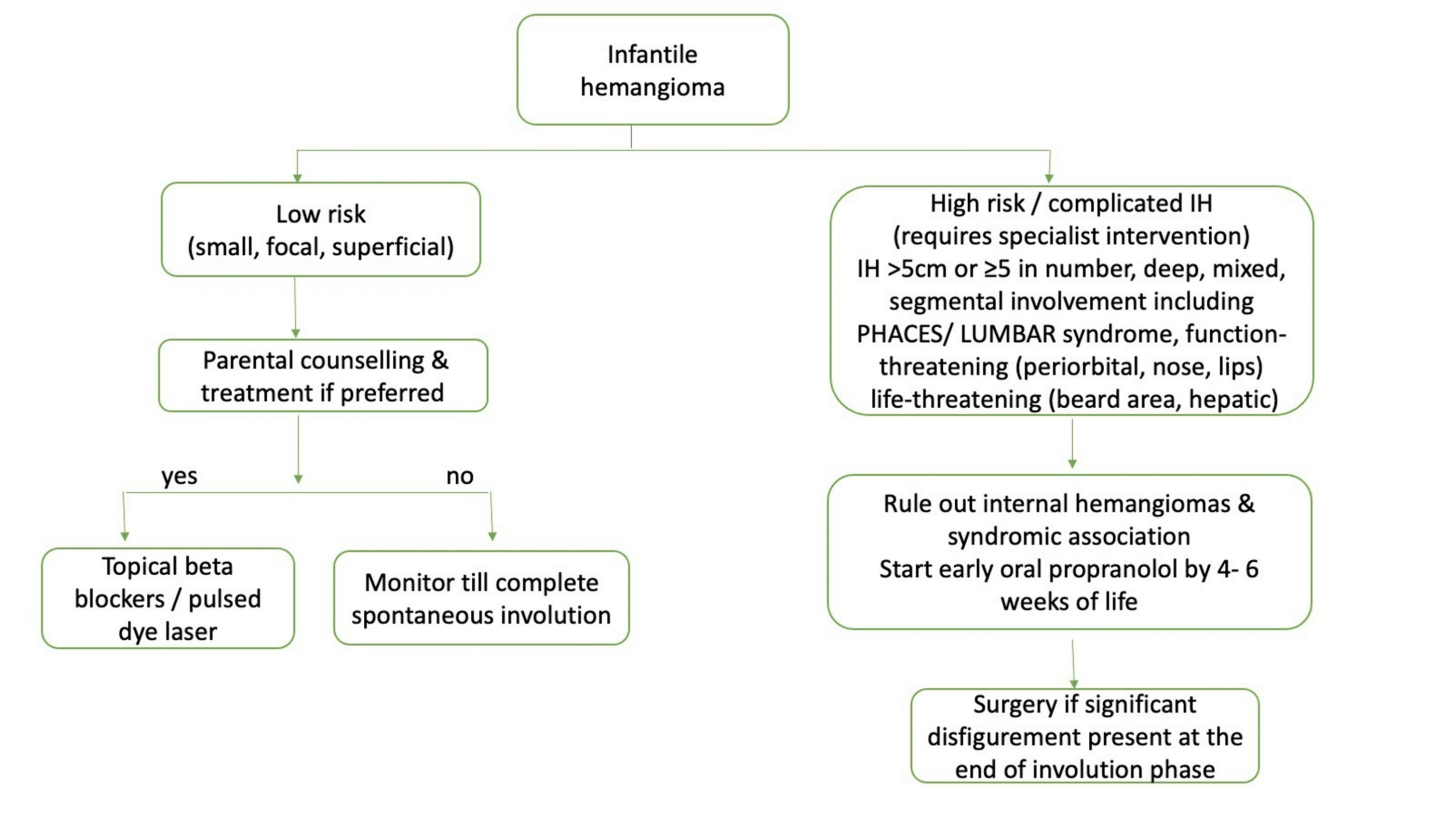
Algorithmic management of infantile hemangioma

We have suggested an algorithmic flowchart that can be followed for appropriate assessment and management of IH (Figure 4). The index case presented to us late in the advanced stage of ulcer plus defaulted treatment for a week and eventually required five months of oral propranolol, following which the hemangioma involuted, albeit leaving a permanent scar.

## Conclusions

IH of the lip can ulcerate and result in the appearance of a cleft lip. It should be managed with oral propranolol as soon as it is diagnosed, unlike many other locations where it can be self-limiting. Treatment should be initiated in the early proliferative phase and continued till the involution phase to minimize the risk of complications. The short-term complications include ulceration, pain, feeding difficulties, and infection, while the long-term sequelae will be permanent disfigurement. The need for surgery will be based on the extent of disfigurement as reconstructive surgery of the lip can be technically challenging due to the uniqueness of the vermilion border of the lip.
